# Granzyme B from mast cells contributes to choroidal neovascularization in a model of wet age-related macular degeneration

**DOI:** 10.3389/fimmu.2026.1710965

**Published:** 2026-02-23

**Authors:** Manjosh Uppal, Amir Hosseini, Khola Bilal, Neilan Tan, Zhengyuan Ai, Wania Khan, Isa Samad, Gurmohit Gill, Hyung-Suk Yoo, Harshini Chakravarthy, Chuan-Hui Kuo, Jeanne Xi, David J. Granville, Joanne A. Matsubara

**Affiliations:** 1 Department of Ophthalmology and Visual Sciences, University of British Columbia (UBC), Vancouver, BC, Canada; 2 Department of Pathology and Laboratory Medicine and ICORD Centre, Vancouver Coastal Health Research Institute, University of British Columbia (UBC), Vancouver, BC, Canada

**Keywords:** angiogenesis, Bruch’s membrane, choroidal sprouting, degranulation, extracellular matrix, outer retina, retinal pigment epithelium, serine protease

## Abstract

**Purpose:**

Wet age-related macular degeneration (AMD) is characterized by choroidal neovascularization (CNV), yet current anti-VEGF therapies are ineffective in many patients. This study investigates the role of mast cell–derived granzyme B (GzmB), a serine protease responsible for the abnormal cleavage of the extracellular matrix in the outer retina.

**Methods:**

Human and mouse choroidal tissues were analyzed for mast cell distribution, GzmB expression, and age-related changes. An ex vivo choroidal sprouting assay (CSA) was used to evaluate the effects of mast cell degranulation and/or stabilization, and the pharmacologic inhibition of GzmB, using tissues from wild-type and GzmB knockout (KO) mice.

**Results:**

Aging increased mast cell accumulation and degranulation in both the human and mouse choroid, leading to elevated GzmB. GzmB KO mice exhibited reduced choroidal sprouting, and exogenous GzmB promoted angiogenesis. Both GzmB inhibition and mast cell stabilization suppressed angiogenic events, confirming GzmB’s role in mast cell-driven angiogenesis.

**Conclusions:**

GzmB is a key mediator of mast cell-induced CNV. Targeting GzmB, either directly or through mast cell stabilization, offers a promising strategy for reducing angiogenesis in a condition such as wet AMD.

## Introduction

Age-related macular degeneration (AMD) is the most common cause of blindness in the elderly population, with an estimated 200 million people affected globally in 2020 (1, 2). This number is expected to rise to nearly 300 million by 2040 due to the aging population. AMD is a multifactorial degenerative disease involving risk factors such as smoking, hypertension, gender, genetics and, most importantly, age. There are two forms of AMD, dry AMD and wet AMD. Geographic Atrophy (GA) or dry AMD is more common, making up 85-90% of all AMD cases, including early and intermediate stages, and is characterized by large and numerous drusen deposits, RPE pigment loss, RPE cell death and attenuation of the choriocapillaris (3–7). Neovascular AMD or wet AMD is characterized by choroidal neovascularization (CNV), where abnormal blood vessels grow into the RPE and retinal layers and leak fluid, leading to hemorrhages and eventually, photoreceptor death and vision loss (2, 8). The main culprit of wet AMD is vascular endothelial growth factor (VEGF), which, when overexpressed, contributes to the neovascularization seen in patients (2, 9). For this reason, VEGF has been the primary therapeutic target for wet AMD; however, VEGF inhibitors, including aflibercept and ranibizumab, have only been effective for 50% patients and largely ineffective for 40-50% of patients; thus, anti-VEGF agents serve as symptomatic treatment rather than a method of prevention (10). Therefore, it remains important to investigate additional mechanisms that lead to wet AMD to allow the development of more effective therapeutics and treatments that will prevent the onset of wet AMD.

Granzyme B (GzmB) is a pro-apoptotic serine protease involved in lymphocyte-mediated killing of target cells (11–13). However, it is now recognized that GzmB can accumulate in the extracellular space where it exerts its proteolytic activity (14, 15). Extracellular GzmB has been linked to numerous chronic inflammatory and autoimmune diseases, including rheumatoid arthritis, age-related and autoimmune skin disorders, atherosclerosis, aneurysm and wound healing (16–21). GzmB is thought to be released into the ECM through leakage from immunological synapses between immune cells and target cells, constitutive release in the absence of target cells, and release from perforin-lacking cells, including mast cells (16, 22–25). Extracellular GzmB cleaves and degrades several known substrates, including cell–cell adhesion proteins, extracellular matrix (ECM) proteins, cytokines, and proteoglycans, which together can promote inflammation, impaired healing and/or pathologic angiogenesis (20, 25, 26). For example, cleavage of fibronectin, an ECM protein and substrate of GzmB, releases sequestered VEGF and increases vascular permeability (17, 26–28). Moreover, the integrity of the outer blood-retinal barrier, which is formed by the tight junctions of the retinal pigment epithelium (RPE) and supported by the Bruch’s membrane (BM), depends on a network of tight junctional and ECM proteins that is also susceptible to extracellular GzmB activity. This degradation further contributes to both angiogenesis and inflammation in neovascular AMD.

Mast cells are tissue-resident immune cells that carry many pro- and anti-angiogenic factors, and their role in angiogenesis has been of recent interest. In the choroid, mast cells have a characteristic distribution where they surround arteries and blood vessels in clusters (29). Cross-sectional staining of the choroid showed that mast cells occupy the Sattler’s and Haller’s layers of the choroidal stroma in aged human eyes, with few in the choriocapillaris (30). Our group demonstrated that GzmB is expressed by both RPE cells and choroidal mast cells, and GzmB is particularly increased in the choroid of older eyes (> 65 years) compared with younger eyes (< 55 years) and in wet AMD eyes with CNV compared with age-matched controls (31). Subsequently, we found that GzmB is released by degranulating choroidal mast cells in an ex-vivo mouse model of AMD (28). We determined that mast cells serve as a source of extracellular GzmB, and, along with other groups, have demonstrated that activation of these mast cells promotes CNV (28, 32). CNV is facilitated through an ECM remodeling mechanism as many GzmB substrates are present in the ECM of the BM and choroid. For instance, GzmB increases the expression of proinflammatory cytokines IL-6 and TGF-β, as well as the chemokine CCL2, while it reduces TSP-1 levels, a known anti-angiogenic factor expressed by the RPE (28). In addition, our group has shown that GzmB deficiency in mice leads to reduced VEGF-A levels in the outer retina and smaller CNV lesion in a laser-induced mouse model of wet AMD (28). These data together highlight the important role of GzmB in modulating the angiogenic response in CNV. We confirmed the accumulation of GzmB in the BM and the presence of GzmB+ mast cells in the mouse and human choroid/sclera (31). Our characterization revealed that these cells exhibit a granular appearance, with GzmB+ choroidal mast cells in the mouse choroid ranging in diameter between 10–25 µm, comparable to those observed in the human choroid. In addition, we reported that initiating mast cell degranulation can effectively increase choroidal sprouting and is facilitated in part by the released GzmB.

In this study, we build upon previous findings by comprehensively characterizing the morphology, distribution, and abundance of GzmB+ mast cells in the choroid of human and mouse eyes. Using the ex vivo choroidal sprouting assay (CSA) as a model of microvessel growth in the outer retina, we investigate how mast cell degranulation and GzmB activity drive CNV. Additionally, we assess whether pharmacological inhibition of GzmB can effectively reduce choroidal sprouting, providing insights into potential therapeutic strategies for wet AMD.

## Methods

### Animals

Animals used in this study followed protocols approved by the University Animal Care Committee at the University of British Columbia, which conform to the guidelines of the Canadian Council on Animal Care, in accordance with the resolution on the Use of Animals in Research of the Association of Research in Vision and Ophthalmology. C57BL/6J (wildtype or WT) and GzmB KO mice on a C57BL/6J background were acquired from Jackson Laboratory (Bar Harbor, ME, USA). Mouse eyes were collected from multiple experimental groups: (1) normal wild-type (WT) mice and GzmB knockout (KO) mice; (2) age-stratified groups including younger WT (<3 months), older WT (>9 months), younger GzmB KO (<3 months), and older GzmB KO (>3 months). Animals will be anesthetized with isoflurane, followed by CO_2_ euthanasia using a gradual fill rate of 20% of the 10L chamber volume per minute, repeated 3–4 times, in accordance with UBC SOP ACC 03-2012 – *Euthanasia of Adult Rodents Using Inhalant Anesthetic, Followed by Carbon Dioxide*. Eyes will then be enucleated and fixed in 4% paraformaldehyde. Fixed globes were embedded in paraffin wax, and sectioned sagittally to obtain cross sections for subsequent immunofluorescence analysis.

### Human samples

This study was approved by the UBC Clinical Research Ethics Board (CREB # H20-02944). Whole-globe post-mortem eyes were obtained from six younger (1 female, mean age 48, range 22–59) and six older donors (3 female, mean age 72, range 71–75) consented for research via the Eye Bank of BC. Upon arrival, eyes were rinsed in PBS, and the neuroretina was separated from the RPE/choroid. Both tissues were stored in PBS at 4 °C. From each RPE/choroid, 3 mm punches were taken from the superior, inferior, and temporal quadrants.

### GzmB and tryptase immunofluorescence in mouse choroid

Slides were deparaffinized and prepared for immunostaining using citrate buffer, 3% H2O2, and 3% NGS. To characterize GzmB positive cells in the choroid as mast cells, primary antibodies against Tryptase (Abcam, ab2378), a mast cell-specific protease, and GzmB (Abcam, ab4095) were added sequentially, and the slides were incubated for 1 hour at RT and left overnight at 4°C for tryptase and for 2 hours at RT the following day for GzmB. After washing the slides, two secondary antibodies were applied, and the nuclei were labelled with DAPI. The slides were mounted using 50% PBS/Glycerol mixture. Confocal microscopy was completed at 20X, 40X or, in some cases, a digital crop factor was applied (80X magnification). Mast cells were imaged whenever they were seen in the choroid.

### GzmB and tryptase staining in human wholemounts

RPE/choroid tissue was punched into 3 mm discs. After antigen retrieval (citrate buffer) and peroxidase blocking (3% NGS), tissues were incubated with anti-tryptase (Abcam ab2378, 1:100, 5 days, 4°C) and anti-GzmB (Abcam ab4095), 1:100, 2 h, RT). After PBS washes, secondary antibodies were applied for 2 h, followed by DAPI (1:500, 10 min). Samples were mounted in 50% PBS/glycerol and imaged using a Zeiss LSM 800 confocal microscope at 4×–80×. Mast cell size (10–20 µm vs. >20 µm) was analyzed at 4×.

### Confocal imaging

Wholemounts and paraffin sections were imaged using a Zeiss LSM 800 confocal microscope with Zen 2.6 software. Z-stacks and orthogonal views were used to assess tissue depth. Central and peripheral regions near the optic nerve were imaged with fixed settings for fluorescence intensity comparison.

### Quantification of labeling

Tissues were analyzed using ImageJ and the following cells were quantified: Mast cells (tryptase+ with nucleus); GzmB+ cells; GzmB+ with nucleus; Double-labeled mast cells (tryptase+, GzmB+, with nucleus) and degranulating mast cells (± GzmB) Two samples per group were excluded due to background staining; the final sample size: n = 4 per group (younger: mean age of 53; older: mean age of 73).

### Bleaching and toluidine blue staining for mouse choroidal mast cells

C57BL/6J and GzmB KO mice were euthanized, and eyes were fixed in 10% buffered formalin. Some eyes were paraffin-embedded, sectioned (4 – 6 mm sagittal), and mounted. Others were processed as wholemounts where an incision was made posterior to the limbus, the globe bisected, lens removed, and neuroretina separated from the RPE/choroid/sclera. Tissue was either cut into quadrants or flattened with relief cuts.

For bleaching, tissue (either dissected or whole globe) was incubated in 10% H_2_O_2_ in PBS at 55 °C for 2.5 h until pigment cleared, then transferred to PBS. Toluidine blue solution (0.1%) was prepared from a 1% stock (Abcam ab146366) in 70% ethanol and 1% NaCl, adjusted to pH 2.0–2.5. Tissues were stained for 5 min, rinsed in PBS (2×2 min), and mounted using: PBS: Glycerol (20:80) for reusable samples. Images were captured using a Nikon Eclipse 80i brightfield microscope. Mast cells were counted at 20× magnification, scanning left-to-right, top-to-bottom in a systematic manner for all sections to eliminate potential bias.

### CSA explants used in toluidine blue staining

To confirm degranulation post-48/80 treatment, Matrigel™-embedded CSA (see below) samples (#354230) were removed and cleaned of residual Matrigel under a dissection microscope. Samples were bleached as above (faster than whole-eye globes, requiring only one round), then stained with toluidine blue and imaged on the Nikon Eclipse 80i.

### CSA sprouting assay

On Day 0, 3-month-old C57Bl/6J WT or GzmB KO mice were euthanized via CO_2_ and cervical dislocation. Eyes were enucleated into ice-cold CSA culture medium with 2× antibiotics (2% Penicillin-Streptomycin). Under a dissection microscope (Nikon SMZ-2B), globes were cleaned, bisected 2 mm posterior to the limbus, and the retina was removed, leaving the RPE/choroid/sclera (“choroid”) intact. Peripheral choroid tissue was sectioned into ~2×1 mm explants or the entire choroidal tissue was sectioned into quarter segments.

Explants were transferred to 30–60 μL of growth factor-reduced Matrigel™ (#354230) or Geltrex™ (#A1413201) in 24-well plates. After solidifying at 37 °C (10–15 min), 500 μL culture medium was added, and plates were incubated at 37 °C, 5% CO_2_ for 48 h before treatment.

On Day 2, media were collected, centrifuged (13,000 rpm, 4 °C, 10 min), and supernatants stored at –80 °C. Wells received fresh media and treatments. Explant growth was imaged at 2× using phase contrast (Nikon H550S, NIS Elements 3.1). Sprouting was quantified using the SWIFT-Choroid macro in ImageJ, where 6600 pixels = 1 mm².

### CSA culture medium

Explants were cultured in Complete Classic Medium (CSC) supplemented with: CultureBoost™ (5 mL per 500 mL CSC); Penicillin/Streptomycin (50 U/mL); Antibiotic-Antimycotic (1×) and Plasmocin™ (5 μg/mL). The culture media was UV-sterilized (15 min) before use, stored in 50 mL aliquots at –20 °C and kept at 4°C once thawed. For dissection, the Penicillin/Streptomycin concentration was quadrupled.

### CSA treatments

On Day 2, the following were added every second day unless noted: HBSS (vehicle control); 48/80 (6.25 μg/mL), a mast cell degranulator; Ketotifen Fumarate (Zaditor^®^, 2.0 μg/mL), a mast cell stabilizer; VTI-1002 (250 μM daily), an extracellular GzmB inhibitor.

VTI-1002, a gift from viDA Therapeutics Inc. (Vancouver, BC), was freshly dissolved in HBSS, aliquoted, and stored at –20 °C. 48/80 stock was prepared fresh each use. Inhibitors (VTI-1002, Ketotifen) were added 30 minutes before 48/80 for optimal effect.

### ELISA

Using the Mouse Mast Cell Tryptase ELISA Kit (CUSABIO, Cat. CSB-E14326m-1), supernatants were added neat into wells and incubated for 1 hour at 37 °C. All wells were washed three times using the manufacturer-provided wash buffer and aspirated. Then, 100µL of HRP-avidin was added to each well and incubated for 1 hour at 37 °C. Wells were washed once again, and 90µL of TMB substrate was added to each well. The plate was then incubated at 37°C for 20 minutes before 50µL of stop solution was added to each well, followed by gentle agitation. Optical density was measured at 540nm with a corrective reading at 450nm using a microplate reader (µQuant, BioTek Instrument Inc.) and KC4 software. A 4-parameter logistic curve was created using the standards and the MyCurveFit plug-in provided by the manufacturer. Optical density measurements were converted to pg/mL, and statistical analysis was completed using GraphPad Prism V9.

### Statistics

All data are expressed as mean ± standard error of the mean. Analysis between the 2 groups was conducted using the Mann-Whitney U test or independent samples t test. By contrast, an analysis between multiple groups was conducted using 1-way analysis of variance, followed by a Tukey *post hoc* test. The statistical analysis was completed using GraphPad Prism (version 9). All CSA experiments were repeated in at least two independent biological replicates with consistent results.

## Results

### Distribution of GzmB+ mast cells in human choroid

**Figure 1:**
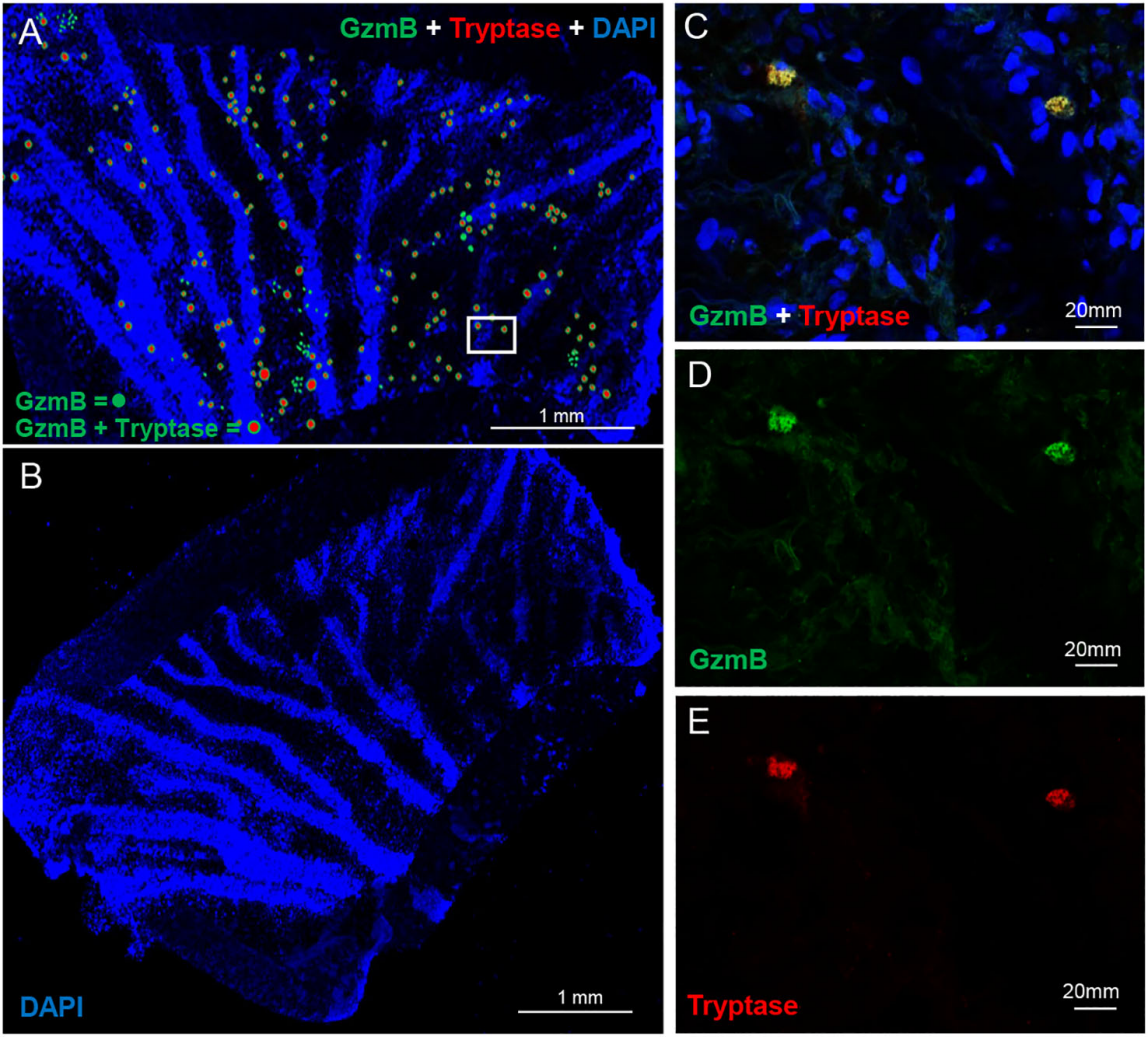
GzmB and Tryptase positive mast cells in human choroidal tissue. Wholemount immunofluorescent labelling of human RPE/choroid tissue revealed the presence and distribution of mast cells expressing Granzyme B (GzmB) and tryptase. **(A)** A low-magnification 4X image of a human choroidal punch shows numerous mast cells distributed throughout the tissue, with many cells co-expressing GzmB (green) and tryptase (red) these have been manual drawn on the correct positions. White box denote zone used for example pictures **(B)** Original DAPI image of human wholemount tissue. Nuclei are labelled with DAPI (blue). **(C–E)** Higher magnification (20X) images show a representative mast cell co-expressing GzmB and tryptase **(C)**, with individual channels displayed for GzmB **(D)** and tryptase **(E)**. These data confirm the presence of GzmB-expressing mast cells in the human choroid and illustrate their granular morphology and perivascular localization.

GzmB+ profiles in the human choroid were explored in a human RPE/choroid wholemount. **Figure 1A** depicts a representative distribution of mast cells positive for both tryptase and GzmB. The mast cells were tryptase+, with a granular appearance and were observed in two size categories, 10-20 µm (shown as smaller circular profiles), and those cells which were >20 µm (shown as larger circular profiles). Approximately 85% of cells were positive for both GzmB and tryptase. However, there were tryptase-/GzmB+ cells and tryptase +/GzmB- cells in the choroid, suggesting that not all mast cells were GzmB+ and not all GzmB+ cells were mast cells. Some signal profiles were smaller than 10 µm and are not shown in the distribution map. GzmB+ mast cells appear to localize near blood vessels and cluster in groups (**Figure 1A**). **Figure 1B** shows the DAPI image of the complete tissue used in **Figure 1A** and outlines the blood vessels in this wholemount. Mast cells were also imaged at higher magnification and displayed cytoplasmic granules typical of a non-degranulation state (**Figures 1C–E**).

### Aging increases GzmB-positive mast cells in the human choroid

**Figure 2:**
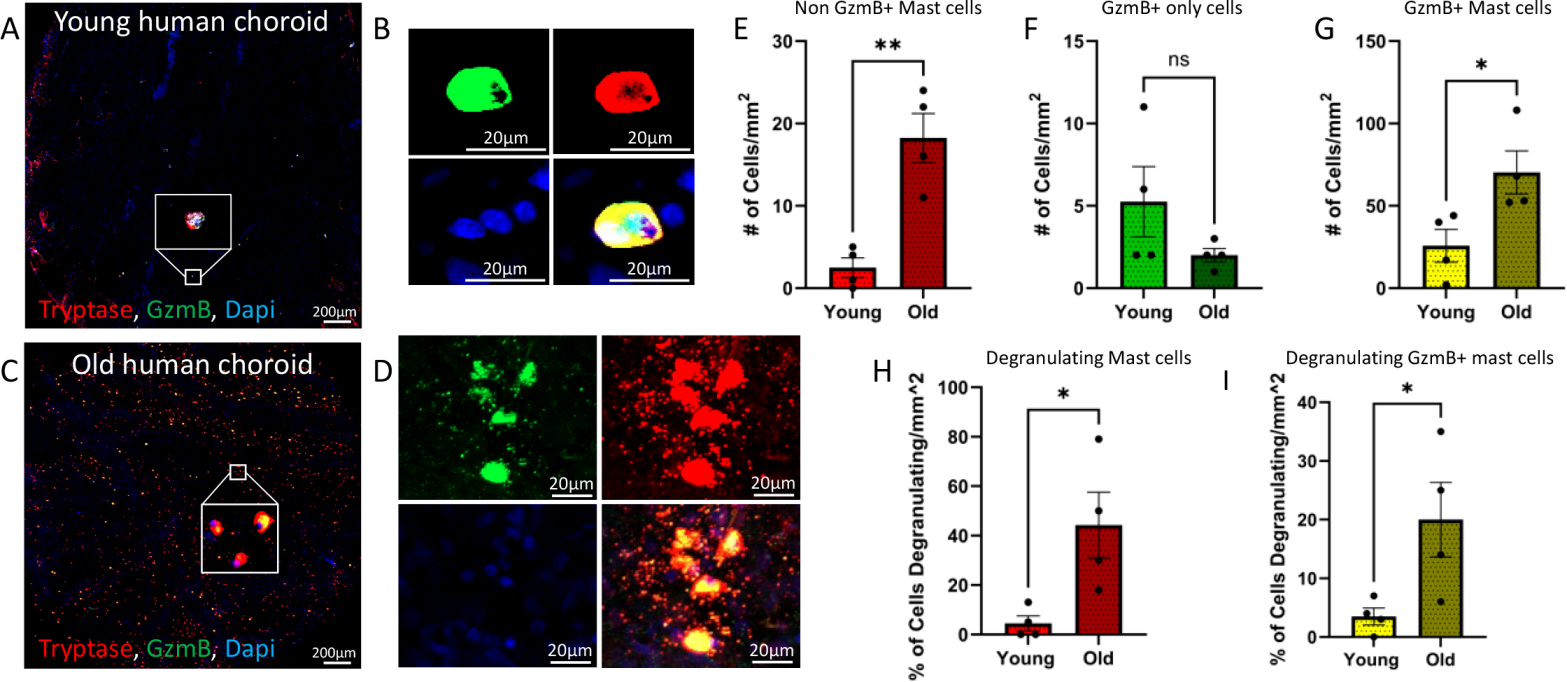
Aging increases GzmB-expressing mast cells and mast cell activation in the human choroid. Immunofluorescent labelling was used to quantify mast cells and their activation state in young (<59) and old (>71) human RPE/choroid tissue. **(A, C)** Representative wholemount images from young and old donor choroidal punches increased numbers of GzmB+ and tryptase+ mast cells in aged tissue. Scale bar = 200µm **(B)** High-magnification view shows a representative condensed, non-degranulated mast cell co-expressing GzmB (green) and tryptase (red), with DAPI-labeled nuclei in the young human choroidal punches. Scale bar = 20µm **(D)** High-magnification views show degranulating mast cells co-expressing GzmB (green) and tryptase (red), with DAPI-labeled nuclei in the old human choroidal punches. Scale bar = 20µm. **(E-I)** Quantification showed a significant increase in tryptase+ mast cells in old vs. young tissue (**E**; p < 0.01) and a corresponding increase in dual-labeled tryptase+ GzmB+ cells (**G**; p < 0.05), total GzmB+ only cell counts did not significantly differ between age groups (**F**; ns). Degranulating mast cells, identified by diffuse GzmB or tryptase signal, were significantly increased in old tissue (**H, I**; p < 0.05). * represents p<0.05. ** represents p<0.01. Unpaired t-tests were used.

Immunostained wholemount RPE/choroid punches from both younger (age <59) and older samples (age >71) demonstrated immunolabeling for both tryptase and GzmB, confirming the presence of mast cells and GzmB in the human choroid amongst the two age groups (**Figures 2A–D**). Following the confirmation of the presence of mast cell and GzmB in the choroid, tryptase+ GzmB- mast cells, tryptase- GzmB+ cells, and tryptase+ GzmB+ mast cells were quantified and compared between younger and older groups. Aging was associated with an increase in both tryptase+ GzmB- mast cells (**Figure 2E**; 2 ± 1 cells/mm² vs. 18 ± 3 cells/mm²; p = 0.0026) and tryptase+ GzmB+ mast cells (**Figure 2G**; 26 ± 10 cells/mm² vs. 70 ± 13 cells/mm²; p = 0.0351). No significant difference was observed in tryptase- GzmB+ cells, the category of cells that had the fewest number in both age groups and represented macrophages, dendritic cells and T cell subsets (**Figure 2F**; 5 ± 2 cells/mm² vs. 2 ± 0.5 cells/mm²; p = 0.1857).

### Aging increases mast cell degranulation in the human choroid

Mast cells release inflammatory substances such as proteases and cytokines into the extracellular space, a process known as degranulation. An age-associated increase in mast cell degranulation was observed (**Figure 2H**; 4.5 ± 3.05% of cells degranulating/mm² vs. 44 ± 13.33% of cells degranulating/mm²; p = 0.0271). Similarly, increased degranulation was noted in GzmB+ mast cells (**Figure 2I**; 3.50 ± 1.44% of cells degranulating/mm² vs. 20.00 ± 6.34% of cells degranulating/mm²; p = 0.0442), suggesting that aging triggers more mast cells to release GzmB in the choroid. Examples of these are shown in **Figures 2B** vs **2D**, where there is evidence of degranulation products (small granules) in the extracellular spaces surrounding mast cells in older donor tissues (**Figure 2D**) compared to the condensed appearance of a representative mast cell in younger tissues (**Figure 2B**). Overall, these data suggest GzmB+ mast cells accumulate and degranulate in aged eyes and represent a mechanism for deposition of extracellular GzmB in the choroid.

### Peripheral localization and GzmB expression profile of choroidal mast cells in mice

**Figure 3:**
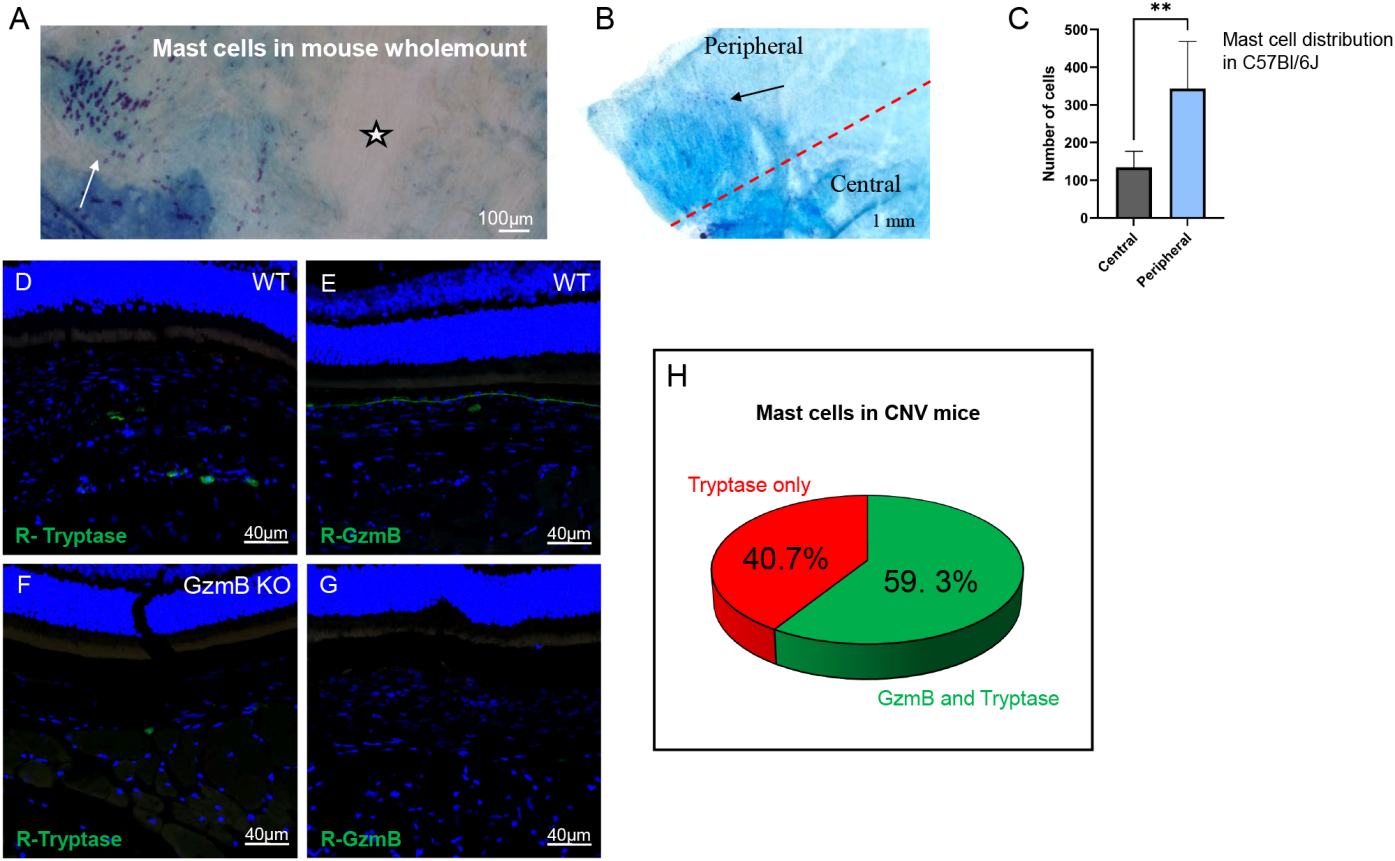
Peripheral localization and GzmB expression profile of choroidal mast cells in WT and KO mice. **(A)** Toluidine blue staining of an albino mouse choroid wholemount showing clustering of mast cells (arrow) and absence in other areas (star) **(B)** Toluidine blue staining of choroidal wholemounts from C57Bl/6J mice shows preferential localization of mast cells (purple granules) to the peripheral choroid (white arrow) as compared to the central region (black star). Scale bar = 100 μm or 1mm. **(C)** Quantification of mast cell distribution confirms significantly higher mast cell density in the peripheral versus central choroid. p < 0.005, unpaired t-test, N = 5. **(D–G)** Immunofluorescence images of choroidal sections stained for tryptase (green, R-Tryptase), Granzyme B (green, R-GzmB), and nuclei (blue, DAPI). WT and GzmB KO mice show comparable tryptase+ mast cell presence in both naïve and CNV lesions. CNV lesions are labeled in WT **(D)** and KO **(G)** conditions. Granzyme B expression is observed in WT but absent in KO tissue (F vs. G). Scale bars = 20 μm. **(H)** Proportion of tryptase+ mast cells that express GzmB in WT CNV mice cross-sections. Approximately 59.3 +/- 16.7% of mast cells are GzmB-positive. N = 3–4 in each group. **p < 0.01. Data are presented as mean ± SEM. Unpaired t-tests were used.

To confirm the presence of mast cells in the mouse choroid, toluidine blue labelling was performed. Toluidine blue labelling on an albino mouse choroid wholemount revealed many mast cells throughout the choroid. **Figure 3A** shows an intact half of a choroid, and mast cells are seen clustering in some regions (white arrow) while vacant in others (star). Toluidine blue+ profiles (mast cells) ranged from dark purple to bright pink and varied due to background color (**Figure 3A**). On average, mast cells ranged from 10µm to 25µm, with some having a more dispersed appearance, making their diameter over 30µm. The color, size, and granular appearance distinguish mast cells from background signals. Counting mast cells in the central and peripheral regions in bleached C57Bl/6J wild-type mouse wholemounts revealed that more mast cells are found in the periphery compared to the central choroidal region (p<0.005) (**Figures 3B, C**).

Previously, GzmB+ mast cells were identified through the colocalization of GzmB with other mast cell markers, such as tryptase and c-Kit, in the mouse choroid (28). To investigate a potential relationship between GzmB+ mast cells and CNV in the mouse, mast cells were examined in mice that had undergone laser-induced CNV. GzmB+ and tryptase+ mast cells were observed in both the choroid and sclera, as shown in **Figures 3D–G**. These findings were compared to GzmB KO, where both KO and wild-type (WT) mice exhibited similar numbers of tryptase+ mast cells. WT mice had an average of 12 tryptase+ mast cells per cross-section, while KO mice had 8 (p = 0.18, n=6). GzmB+ mast cells were quantified in WT cross-sections adjacent to those labeled with tryptase, and approximately 59.3% +/- 16.7% (**Figure 3H**) of the mast cells contained GzmB in mouse cross-sections.

### Age-associated mast cell accumulation in the mouse choroid

**Figure 4:**
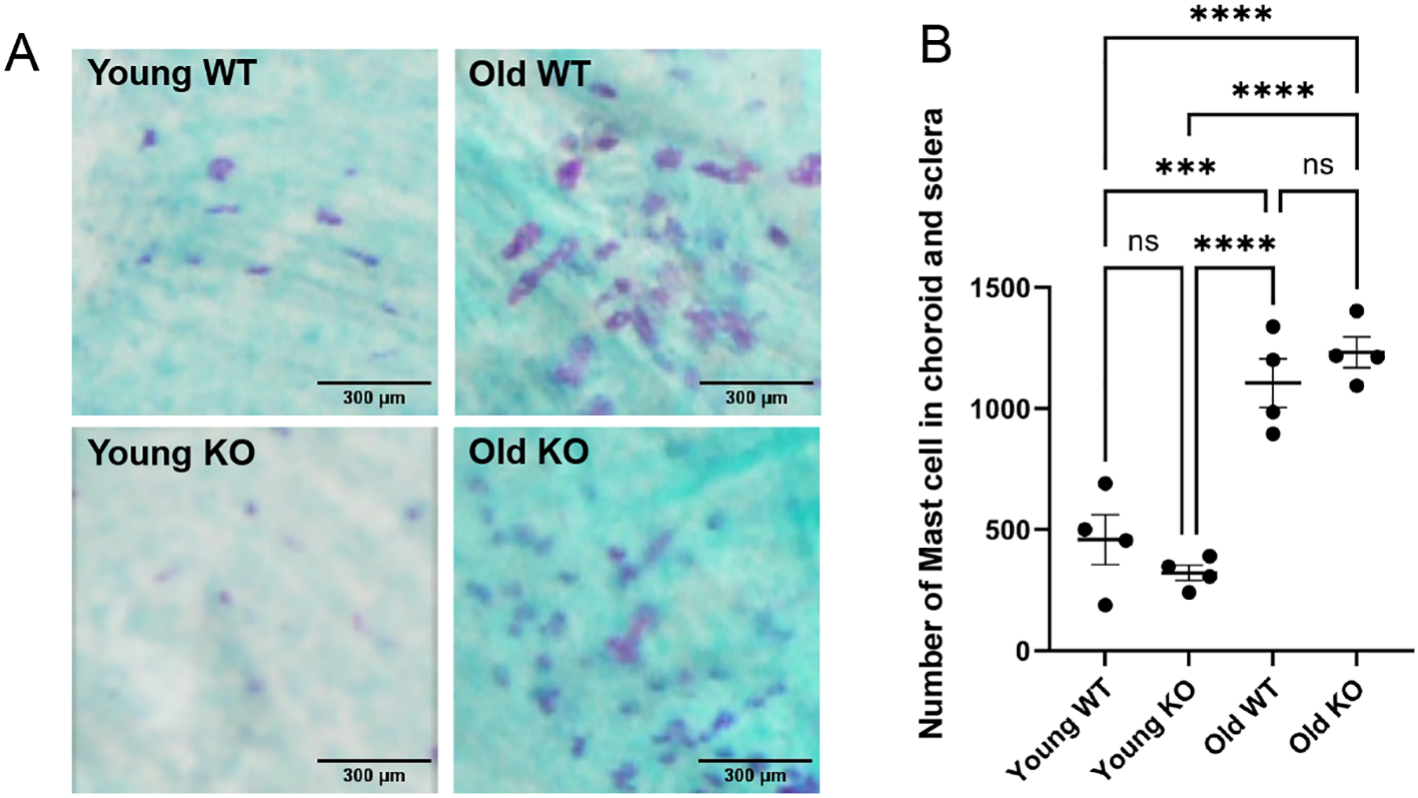
Age-associated mast cell accumulation in the mouse choroid occurs in both WT and GzmB KO mice. Toluidine blue labelling was performed on choroid/sclera wholemounts from young and old C57Bl/6J WT and GzmB KO mice to assess mast cell abundance with age and genotype. **(A)** Representative images from young WT and KO animals show sparsely distributed mast cells (purple granules), whereas tissue from old WT and KO animals revealed numerous, densely packed mast cells (left panels). **(B)** Quantification of mast cells across wholemounts demonstrated a significant increase in mast cell number with age in both WT and KO mice (****p < 0.0001), with no significant difference observed between genotypes at either age. (***p<0.001). N = 6. Young = less than 3 months, Old = older than 12 months. ANOVA with Tukey’s *post hoc* test was used.

To determine if GzmB deficiency affects mast cell population in the mouse choroid, we investigated the total number of mast cells in WT vs GzmB KO mice at baseline. Toluidine blue labelling was performed on choroidal wholemounts from younger (<3 month) and older (>9 month) WT and GzmB KO mice. Mast cells were sparsely distributed in the choroid and sclera of younger WT and GzmB KO animals. In contrast, mast cells were markedly increased in both older WT and GzmB KO choroid and sclera (**Figure 4A**), with numerous darkly stained, granular profiles evident throughout the sections. Quantification of mast cells revealed a significant increase in the number of mast cells in older animals, regardless of genotype (p < 0.0001) (**Figure 4B**). There was no significant difference in mast cell numbers between WT and KO animals at either age, suggesting that GzmB deficiency does not alter mast cell accumulation in the outer retina with age. Overall, these data indicate that mice also exhibit age-dependent accumulation of GzmB+ mast cells, similar to what is observed in the human tissues.

### Choroidal sprouting in RPE/Choroid explants is reduced in GzmB KO and exogenous GzmB restores choroidal sprouting

**Figure 5:**
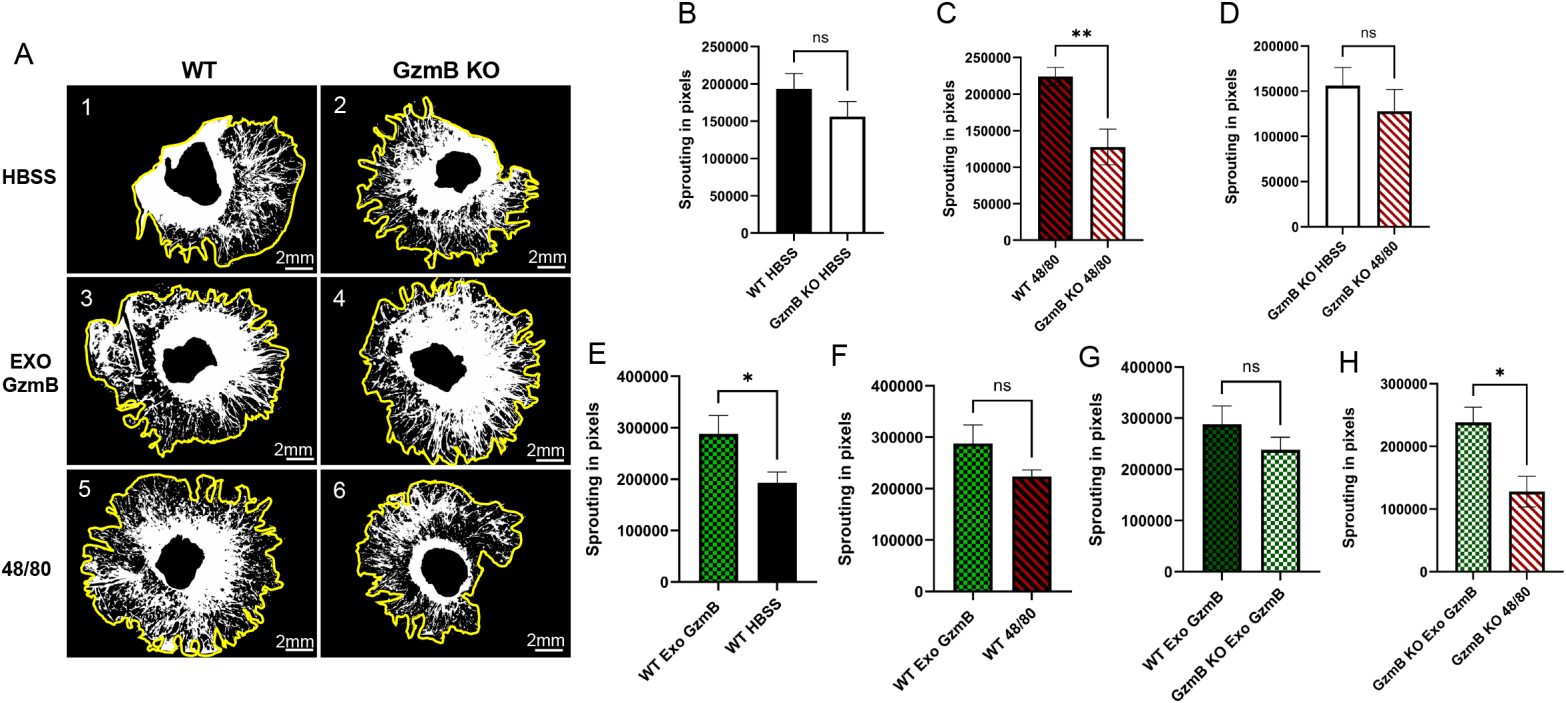
Granzyme B promotes choroidal sprouting: reduced angiogenesis in GzmB KO mice and restoration by exogenous GzmB. **(A)** (1-6) Day 8 Representative images of choroidal sprouting under different experimental conditions. Yellow outline indicates sprouting area, middle indicates explant. **(B–D)** Quantification of sprouting in wild-type (WT) and GzmB knockout (KO) mice treated with HBSS or mast cell degranulator compound 48/80. No significant difference in baseline sprouting between WT and GzmB KO mice **(B)**, but a significant reduction in sprouting was observed in GzmB KO mice compared to WT following 48/80 treatment **(C)**. No difference was observed in KO mice treated with 48/80 versus HBSS **(D)**. **(E–H)** Effect of exogenous GzmB (Exo GzmB) on choroidal sprouting. Exogenous GzmB significantly increased sprouting in WT mice compared to HBSS control **(E)**, but did not enhance sprouting beyond that induced by 48/80 **(F)**. In GzmB KO mice, sprouting with Exo GzmB was not significantly different from WT **(G)**, but significantly higher than 48/80 treatment alone **(H)**, indicating partial rescue. All data are presented as mean ± SEM. N = 6 explants per condition. Statistical analysis was performed using unpaired t-tests. *p < 0.05, **p < 0.005; ns = not significant.

Previously, Obasanmi et al. (26) reported that pharmacological inhibition of GzmB leads to decreased choroidal sprouting; therefore, the impact of a GzmB deficiency on choroidal sprouting was investigated using RPE/choroid explants harvested from GzmB KO animals (**Figure 5A**). Day 8 sprouting, shown in **Figure 5B** (comparing 5A-1 and 5A-2) reveals similar basal-level growth in WT compared to GzmB KO explants. In contrast, 48/80 treated WT explants exhibited more sprouting compared to 48/80 treated GzmB KO explants as shown in **Figure 5C** (comparing 5A-5 and 5A-6) (p<0.005). GzmB KO controls (HBSS) exhibited similar levels of sprouting as GzmB KO treated with 48/80 (**Figure 5D**, comparing 5A-2 and 5A-6), suggesting that, in the absence of GzmB, mast cell degranulation does not induce a significant angiogenic response. Next, WT explants stimulated with 100 nM of exogenous GzmB induced increased sprouting relative to HBSS alone (p<0.05) (**Figure 5E**, comparing 5A-1 and 5A-3) consistent with our earlier findings (26). Next, WT explants stimulated with GzmB showed sprouting that was not significantly greater than explants treated with 48/80 (**Figure 5F**, comparing 5A-3 and 5A-5). GzmB KO explants treated with exogenous GzmB had similar levels of sprouting compared to WT explants treated with exogenous GzmB (**Figure 5F**, comparing 5A-3 and 5A-4). Finally, in contrast to WT explants, GzmB KO explants treated with exogenous GzmB exhibited significantly more sprouting than GzmB KO explants treated with 48/80, suggesting that exogenous GzmB has a similar pro-angiogenic effect as mast cell degranulation but only when GzmB is present within mast cells (**Figures 5G, H**, comparing 5A-4 and 5A-6) (p<0.05).

### Pharmacological inhibition of GzmB exerts similar anti-angiogenic effects as GzmB deficiency

**Figure 6:**
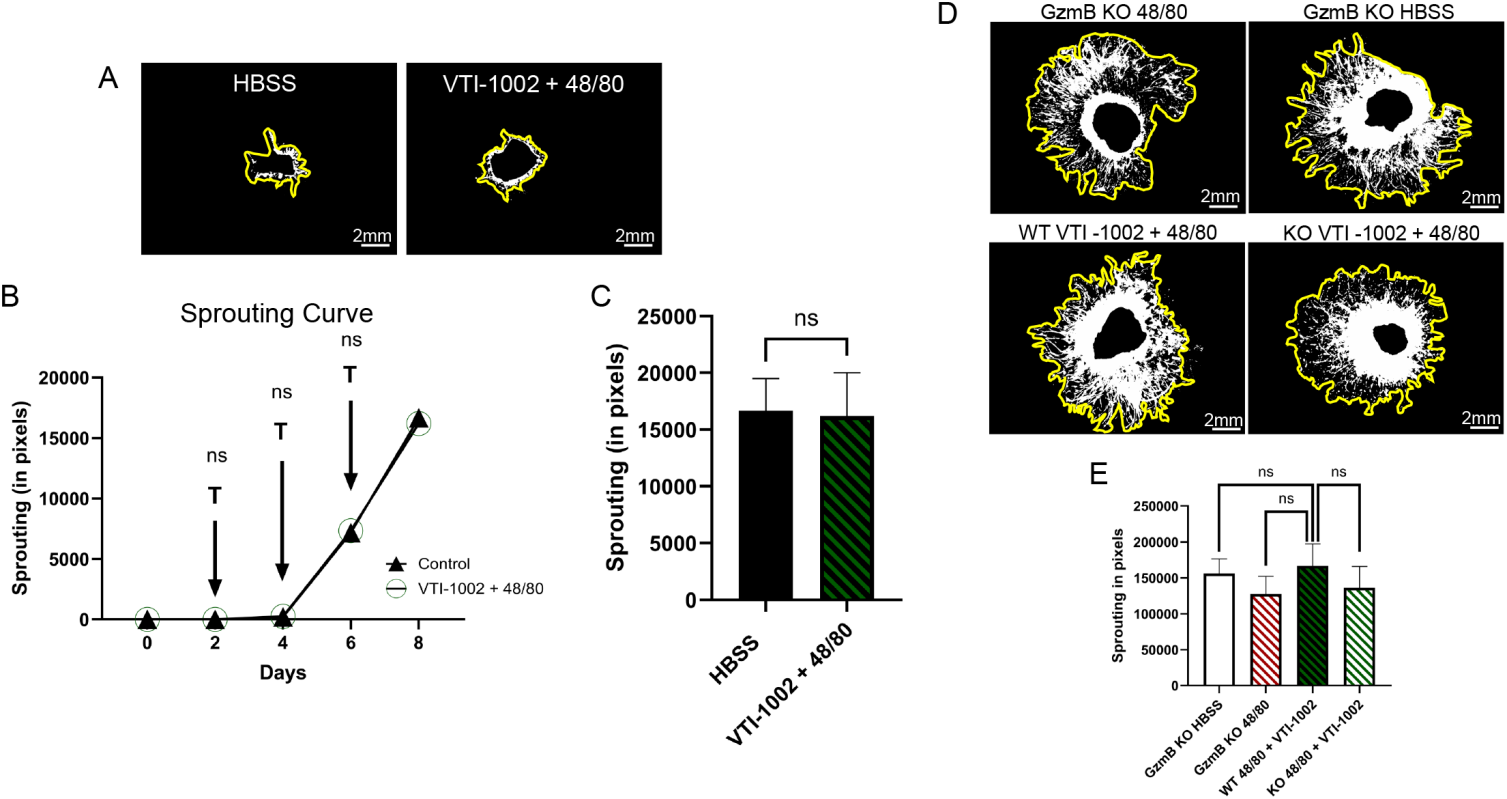
Pharmacologic inhibition of Granzyme B mimics GzmB knockout and abrogates mast cell–induced choroidal sprouting. **(A, D)** Day 6 and Day 8 representative explant images showing choroidal sprouting under the indicated conditions respectively. Yellow outline indicates sprouting area, middle indicates explant. **(B)** Time-course analysis of choroidal sprouting in explants treated with mast cell degranulator (48/80) in the presence or absence of the GzmB inhibitor VTI-1002. 48/80 was applied on days 2, 4, and 6 (arrows marked “T”) but VTI-1002 was added daily. No significant differences in sprouting were observed at any time point. **(C)** Quantification of sprouting in WT explants treated with 48/80 and VTI-1002 compared to HBSS controls shows no significant difference under GzmB inhibition. **(E)** Comparison of sprouting between WT and GzmB KO explants both treated with 48/80 and VTI-1002 shows no significant difference, further supporting that pharmacologic GzmB inhibition is similar to GzmB knockout explant sprouting. Data are presented as mean ± SEM. N = 3–6 per condition. Statistical analysis was performed using unpaired t-tests. ns = not significant.

In our earlier study (26) we reported that 48/80 caused increased choroidal sprouting in the WT, while the introduction of a small-molecule GzmB-specific inhibitor (VTI-1002), significantly reduced choroidal sprouting. Here, we compared these results to explants from GzmB KO mice. After Day 6, there was no significant difference in the amount of sprouting from the 48/80 + VTI-1002 treated GzmB KO explants compared to the GzmB KO control (HBSS) treated explants, as shown in **Figure 6A**. This was maintained at Day 8, where the control (HBSS) treated explants continued to sprout at the same rate as 48/80 + VTI-1002 treated explants (**Figures 6A–C**) suggesting that the GzmB inhibition reduces the sprouting back to control levels. In a following experiment, this set of treatment (48/80 + VTI-1002) was compared to GzmB KO explants also treated with (48/80+VTI-1002) and GzmB KO explants treated with HBSS or 48/80, and similar levels of sprouting were observed in all groups (**Figures 6D, E**), suggesting that pharmacological inhibition of GzmB mimics the anti-angiogenic effects of GzmB KO (n=6).

### Mast cell stabilizer prevents degranulation and reduces sprouting in CSA

**Figure 7:**
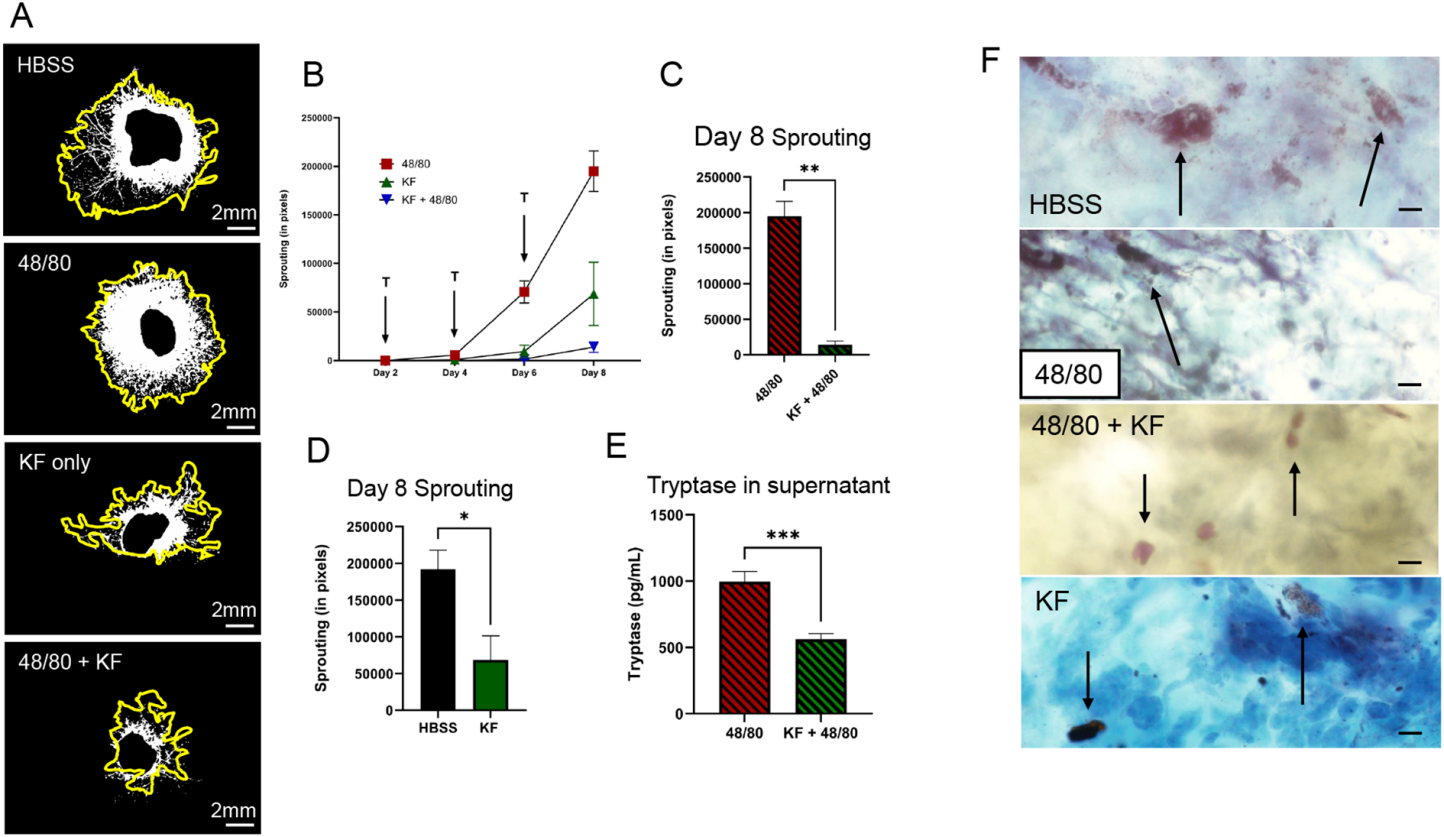
Mast cell stabilizer prevents mast cell degranulation and reduces sprouting. **(A)** Day 8 representative explant images showing choroidal sprouting under the indicated conditions. Yellow outline indicates sprouting area, center black outline indicates explant. **(B)** Time-course analysis of choroidal sprouting in explants treated with mast cell degranulator (48/80) in the presence or absence of the KF. 48/80 was applied on days 2, 4, and 6 (arrows marked “T”) alongside KF. Significant differences in sprouting were observed at 4, 6 and 8 days. **(C, D)** Quantification of sprouting in WT explants treated with 48/80 +/- KF or just KF vs control shows significantly more sprouting when the mast cell degranulator is not present. (N=3). **(E)** Tryptase ELISA on CSA supernatants from indicated conditions. 48/80 treated explant supernatants contain significantly more tryptase than KF treated supernatant samples. (N=3). **(F)** Bleached CSA choroids stained with Toluidine blue after the treatments indicated. Arrows point to mast cells (or degranulated mast cells). Scale bar represents 20µm. Data are presented as mean ± SEM. N = 3 per condition. Statistical analysis was performed using unpaired t-tests. *p<0.05, **p<0.01, ***p<0.001, ns = not significant.

Previously, we demonstrated mast cell degranulation caused an increase in choroidal sprouting (28). We reported that ketotifen fumarate (KF), a mast cell stabilizer, applied in conjunction with 48/80, causes a significant decrease in the choroidal sprouting response (28). The representative picture and graph from Day 8 illustrate this drastic reduction in sprouting, which was the case throughout the extent of the CSA experiment, as shown in **Figures 7A, B**. Next, we explored the effect of KF alone compared to vehicle (HBSS) treatment and found a reduction in sprouting in the KF group **Figure 7D** (p<0.05, n=4). Given that this reduction was not as significant in magnitude as the 48/80 vs 48/80 + KF comparison as seen in **Figure 7C**, there may be a baseline level of mast cell activation occurring in the HBSS group that the KF was able to suppress.

To confirm that the addition of KF at 2.5 µg/mL prevented mast cell degranulation, a Tryptase ELISA was performed. The results of the ELISA using supernatant samples collected 48 hours following treatment revealed increased tryptase content in 48/80 treated samples compared to KF + 48/80 treated samples where there is a significant 2-fold increase in tryptase in the 48/80 treated samples compared to the KF + 48/80 treated samples (**Figure 7E**). This was explored further using Toluidine blue staining on CSA explants after the CSA experiment had concluded. Toluidine blue staining revealed compact, non-degranulating mast cells in KF or KF + 48/80 treated explants (**Figure 7F**). Mast cells in 48/80 treated explants were non-distinguishable as a cloud of granules was seen throughout the explant, suggesting mast cell degranulation had occurred. Explants treated as control (HBSS) showed mast cells in both degranulated and non-degranulating states which supports the baseline mast cell activity observed in the KF-treated groups. Overall, the ELISA and Toluidine blue staining data suggest that KF successfully stabilized mast cells in the CSA compared to 48/80 or HBSS treatments.

## Discussion

This study provides evidence that mast cell-derived GzmB, acts as a critical modulator of angiogenesis in an explant model of wet AMD. Extracellular GzmB activity promotes ECM cleavage, promoting the breakdown of the outer blood retinal barrier, subsequently promoting pathologic angiogenesis. This process may be further facilitated by GzmB-induced release of pro-angiogenic factors such as VEGF, as well as the degradation of endogenous anti-angiogenic proteins like thrombospondin-1 (TSP-1) (26, 28, 33).

Mast cells are tissue-resident immune cells that store and release a wide range of biologically active mediators, including amines, proteases, enzymes, and cytokines, and play well-established roles in allergic and inflammatory responses (34). Their phenotype and function are highly dependent on the local tissue environment, making it essential to study mast cells in the specific biological context in which they act (35). In the context of AMD, mast cells have garnered increasing interest due to their ability to modulate angiogenesis and inflammation (36). Prior studies have shown that mast cells accumulate and undergo degranulation in the choroid of AMD patients, suggesting their active involvement in both dry and wet AMD pathogenesis (28, 30, 31, 37, 38). Positioned near the choroidal vasculature, mast cells contribute to neovascularization through the release of proangiogenic mediators such as histamine, VEGF, GzmB, tryptase, and other proteases (37, 39, 40). For example, tryptase can directly promote endothelial cell proliferation and matrix remodeling, facilitating choroidal neovascular membrane formation (39). In addition, GzmB mediated cleavage of fibronectin, releases sequestered VEGF and increases vascular permeability (17, 26, 27). Recent evidence suggests that mast cell activation is enhanced by oxidative stress and may be modulated by TSP-1 (41–43). Dysregulation of TSP-1, either through reduced expression or proteolytic cleavage by GzmB, can shift the ocular microenvironment toward a proangiogenic state, amplifying mast cell-mediated vascular responses (33, 44, 45). While mast cells are increasingly implicated in the angio-inflammatory processes of wet AMD, critical questions remain regarding their temporal activation, interaction with endothelial cells, and regulation by TSP-1 and complement components *in vivo*. Future studies should aim to clarify these mechanisms and evaluate the efficacy of mast cell stabilizers as adjunct or alternative therapies to current anti-VEGF treatments.

In the present study, we characterized the morphology, localization, and age-related changes of GzmB-expressing mast cells in both human and mouse choroid. In our previous work, we mapped the distribution of GzmB-positive cells in the human choroid but were unable to definitively identify their cellular source (31). Here, by co-labelling for tryptase, a mast cell–specific protease, we confirmed that many GzmB-positive profiles are indeed mast cells (28, 31). These cells exhibited a granular appearance, localized predominantly around blood vessels, and ranged in size from 10–25 µm. In older eyes, approximately 80% of tryptase-positive mast cells also co-expressed GzmB, indicating that mast cells are a major source of GzmB in the choroid.

AMD is a multifactorial disease influenced by genetic, dietary, and environmental factors; however, age remains the strongest risk factor for disease development. Our data demonstrate that GzmB-containing mast cells are present across age groups but are significantly more abundant in older individuals. Moreover, the number of mast cells lacking GzmB expression also increased with age, suggesting a broader, age-dependent accumulation of mast cells in the choroid. While previous studies have demonstrated increased mast cell numbers in AMD-affected eyes, our findings show that aged choroids exhibit both a higher density of mast cells and an increased proportion of degranulating mast cells. This suggests that aging not only promotes choroidal mast cell accumulation but also enhances their activation, thereby facilitating the release of granule contents including GzmB (30).

We next investigated whether similar patterns were observed in mice. Using toluidine blue staining and immunolabeling, we confirmed that mast cells in mice were enriched in the peripheral choroid and sclera and often appeared in clusters, and that the majority of tryptase+ mast cells also expressed GzmB in WT mice after laser-induced CNV, confirming that homeostatic and CNV conditions display subsets of choroidal mast cells that contain GzmB.

Importantly, GzmB KO mice had similar numbers and distributions of tryptase+ mast cells as WT controls. This indicates that GzmB is not required for mast cell development, recruitment, or maintenance in the choroid, but rather serves as a functional effector released upon mast cell activation.

Aging was also associated with a significant increase in mast cell number in both WT and GzmB KO animals. This age-related increase in mast cells occurred regardless of genotype, further supporting the conclusion that GzmB is not involved in age-dependent choroidal mast cell recruitment but is more likely to act an effector molecule under inflammatory or pathological conditions.

To assess the functional role of GzmB in choroidal angiogenesis, we used CSA, an explant model of CNV (46). Under basal conditions, GzmB KO explants exhibited similar levels of sprouting compared to WT explants. When mast cell degranulation was induced using compound 48/80, WT explants showed a robust increase in sprouting, whereas GzmB KO explants did not exhibit an angiogenic response. This suggests that mast cell degranulation promotes choroidal angiogenesis in a GzmB-dependent manner. Exogenous addition of GzmB rescued the sprouting phenotype in GzmB KO explants and further increased sprouting in WT explants. The magnitude of this effect was similar to that in WT explants treated with 48/80. These data further support the prior literature on GzmB as an important mediator of the angiogenic effects of mast cell degranulation in the choroid (28, 31, 33). The exogenous GzmB was applied to the supernatant in our experiments. Earlier studies showed that exogenous GzmB had similar activity to endogenous GzmB in its ability to cleave ECM proteins; however, its localization within the tissue may be important and needs to be investigated further (26, 28). Future experiments are also needed to explore the possibility of an additive effect when 48/80 and exogenous GzmB are applied together and if these findings are replicable *in vivo* with laser-induced CNV.

To determine whether targeting GzmB could serve as a viable therapeutic strategy, we applied VTI-1002, a small-molecule inhibitor to the CSA model (28, 47). VTI-1002 and a first-in-class, highly potent, selective inhibitor of human Granzyme B (GzmB) with Ki of 4.4 nM, that exhibits strong selectivity for GzmB over caspases 3-10, cathepsin G, and neutrophil elastase; VTI-1002 is nearly 20 times more potent against human GzmB than Ac-IEPD-CHO (Ki=80 nM), and exhibits potent inhibition against mouse GzmB (IC50 = 179 nM); improves remodeling in a murine model of impaired burn wound healing (48–51).

We previously showed that treatment with VTI-1002 significantly reduce 48/80-induced sprouting in WT explants, and here we found that it reduced sprouting levels down to the baseline (28). Notably, when VTI-1002 was applied to GzmB KO explants, no additional reduction in sprouting was observed, confirming that the inhibitor acts specifically through GzmB and not via off-target effects. These findings mirror our prior observations with GzmB KO mice and demonstrate that pharmacologic inhibition of GzmB can effectively suppress choroidal angiogenesis driven by mast cell activation.

To further validate the role of mast cell degranulation in GzmB-mediated angiogenesis, we investigated the effect of mast cell stabilization using KF, a well-characterized mast cell stabilizer that prevents granule release (52, 53). In the CSA, explants treated with 48/80 alone exhibited significantly enhanced vascular sprouting, consistent with mast cell activation and mediator release. However, co-treatment with KF effectively suppressed this enhanced sprouting, bringing the response down below baseline levels. Interestingly, KF suppressed sprouting in the vehicle treatment (HBSS), suggesting there may be some baseline mast cell activation that may have been triggered by tissue dissection. Other groups have explored the use of KF in treatment of CNV and found similar powerful anti-angiogenic effects (32).

This KF-mediated mast cell suppression was further supported by biochemical and histological evidence. ELISA performed on CSA supernatants showed reduced tryptase levels in KF-treated explants, indicating diminished degranulation. In parallel, toluidine blue staining revealed compact, intact mast cells in KF and KF + 48/80–treated tissues, in contrast to the diffuse granule patterns observed in 48/80-only conditions. These findings confirm that KF prevented mast cell degranulation, including the release of GzmB, and thereby blocked downstream angiogenic signaling. Importantly, KF treatment alone (in the absence of 48/80) also modestly reduced baseline choroidal sprouting, suggesting that tonic mast cell activation may contribute to basal angiogenic tone in the choroid. Together, these results demonstrate that mast cell degranulation is a prerequisite for GzmB release and angiogenic remodeling and that pharmacological stabilization of mast cells is an effective upstream strategy to suppress this pathway.

Consistent with our previous findings, mast cell activation robustly enhanced choroidal sprouting in the CSA, while mast cell stabilization markedly suppressed this response (28). These effects were mirrored at the biochemical level by reduced tryptase levels and confirmed histologically by the presence of compact, non-degranulating mast cells in KF-treated explants. These results align with the findings in previous literature, where intraperitoneal injection of KF suppresses CNV formation in the laser-induced mouse model of CNV, and there is a proposed pro-angiogenic role for tryptase in choroidal pathology (54). Together, our data support the hypothesis that mast cells are a critical cellular source of angiogenic mediators, including GzmB and tryptase, and reinforce the potential of stabilizing mast cells or selectively targeting their secretory products as upstream therapeutic strategies in wet AMD. While suppression of mast cell degranulation may be a viable strategy in reducing an angiogenic response, mast cells function as a vital immune modulator, and questions remain whether suppression may result in unwanted side effects. Although not explored in this study, mast cells and GzmB have been linked to subretinal fibrosis, a common complication in wet AMD patients after anti-VEGF treatment (26, 33, 47, 55–59). Pharmacological inhibition of GzmB specifically may serve as a valuable strategy to suppress the pro-angiogenic and pro-fibrotic processes underlying CNV formation and subretinal fibrosis.

Taken together, our data support a model in which aging promotes mast cell accumulation and activation in the choroid, leading to increased release of GzmB into the extracellular space. GzmB then acts on the extracellular matrix to remodel the tissue environment and promote pathological angiogenesis characteristic of wet AMD. Both genetic ablation and pharmacologic inhibition of GzmB reduce this angiogenic response, identifying GzmB as a key mediator of mast cell-driven CNV. GzmB is also found in RPE cells within the outer retina; however future experiments are needed to determine how RPE-derived GzmB contributes to wet AMD.

Given that anti-VEGF therapies do not benefit all patients and primarily offer symptomatic relief, targeting upstream effectors like GzmB may offer a novel approach to modulating the disease process itself. The findings from this study suggest that GzmB inhibition, either directly or through mast cell stabilization, decreases angiogenesis, an important component in wet AMD.

## Data Availability

The authors declare that all the data supporting the findings of this study are available within the main text or the **Supplemental Material**.

## Supplementary material

Supplementary Figure 1 **(A)** Day 8 representative explant images showing choroidal sprouting under the indicated conditions. Yellow outline indicates sprouting area, middle indicates explant. **(B)** Quantification of sprouting in WT explants treated with 48/80 at 3 dosages (low, 6.25 µg/mL) (Medium, 12.5 µg/mL) (High, 25.0 µg/mL) and trends toward low and medium dosage causing more sprouting compared to when the mast cell degranulator is not present. (N = 3). **(C)** ELISA on CSA supernatants testing for levels of Tryptase. 48/80 treated explants exhibit the most tryptase in supernatant relative to controls and gel controls (N = 3). *p<0.05, **p<0.01. Mann-Whitney U tests used.

Supplementary Figure 2 BSA done on 48/80 treated explant supernatant from CSA compared to HBSS controls. (N = 3). Mann-Whitney U test used.

Supplementary Figure 3 Negative control of wholemount human RPE choroid tissue. **(A–D)** Representative images showing Alexa 546, Alexa 488, merged, and DAPI channels, respectively, in the absence of primary antibodies. For this negative control, no primary antibodies were added. Instead, during the primary antibody incubation step, the tissue was incubated in blocking buffer alone for the same duration as the primary antibody incubation used for the stained tissues. All other staining steps, reagent concentrations, wash conditions, and imaging parameters were identical to those used for the primary stained tissues, as outlined in the Methods. Scale bar: 50 µm.
