# Supplementary figures and images for: Granzyme B from mast cells contributes to choroidal neovascularization in a model of wet age-related macular degeneration
Source: Front Immunol. 2026 Feb 23;17:1710965. doi: 10.3389/fimmu.2026.1710965 (PMC12968239; doi:10.3389/fimmu.2026.1710965)

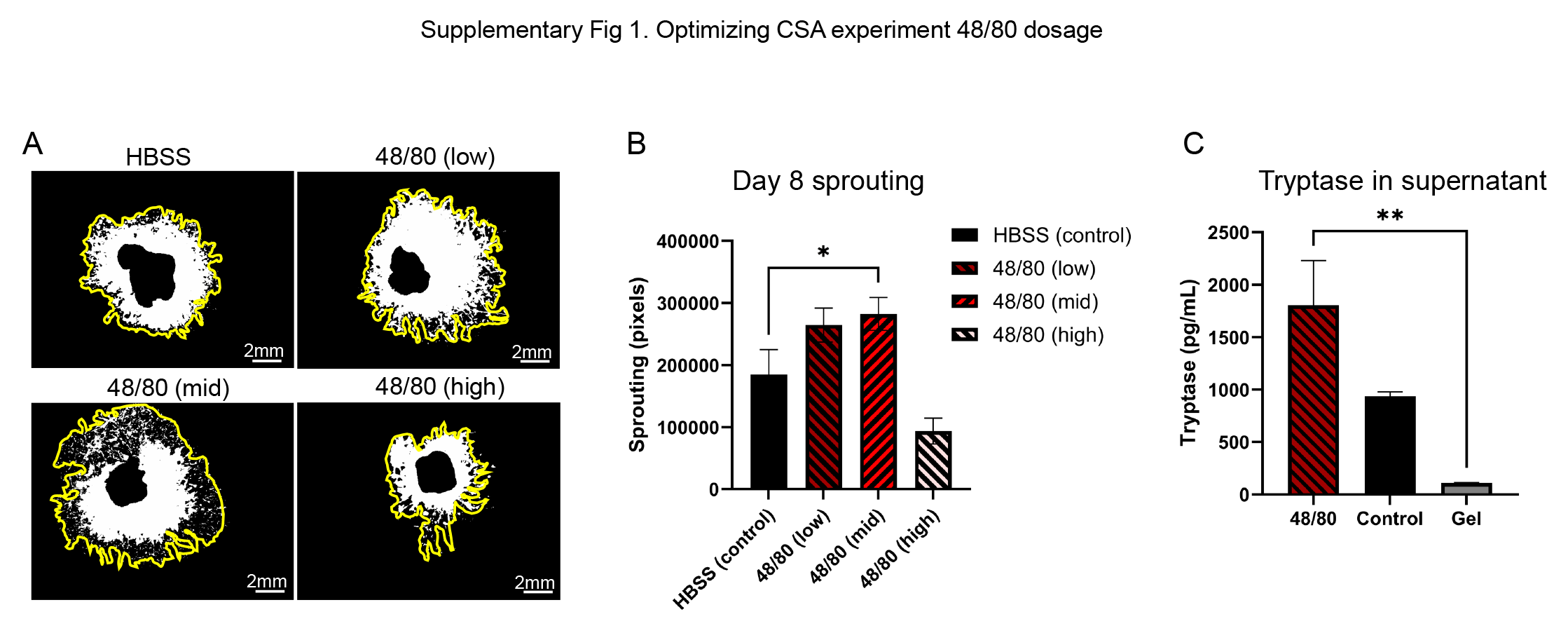

Supplement: Supplementary Figure 1 — (A) Day 8 representative explant images showing choroidal sprouting under the indicated conditions. Yellow outline indicates sprouting area, middle indicates explant. (B) Quantification of sprouting in WT explants treated with 48/80 at 3 dosages (low, 6.25 µg/mL) (Medium, 12.5 µg/mL) (High, 25.0 µg/mL) and trends toward low and medium dosage causing more sprouting compared to when the mast cell degranulator is not present. (N = 3). (C) ELISA on CSA supernatants testing for levels of Tryptase. 48/80 treated explants exhibit the most tryptase in supernatant relative to controls and gel controls (N = 3). *p<0.05, **p<0.01. Mann-Whitney U tests used. [file Image1.tif]

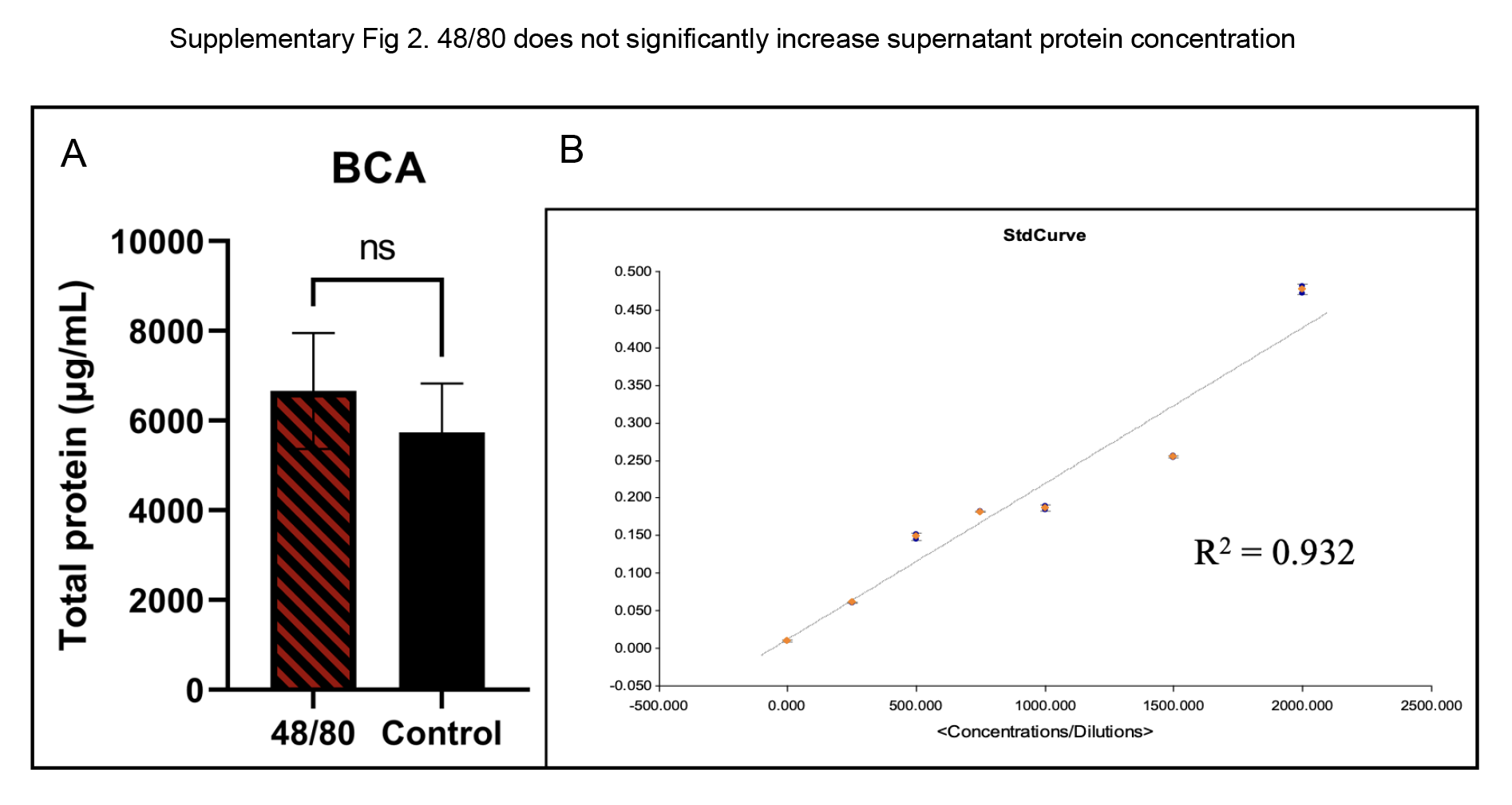

Supplement: Supplementary Figure 2 — BSA done on 48/80 treated explant supernatant from CSA compared to HBSS controls. (N = 3). Mann-Whitney U test used. [file Image2.tif]

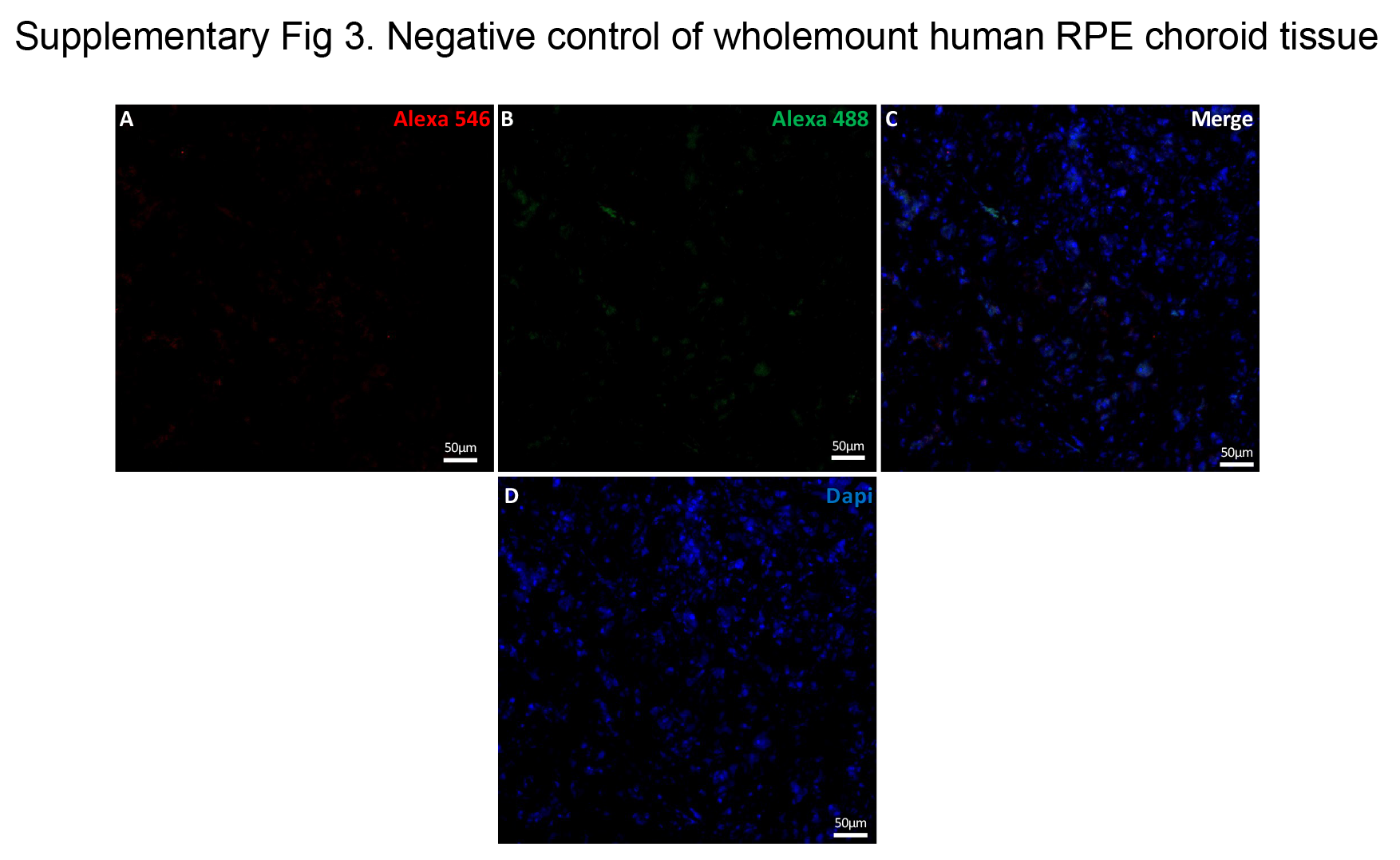

Supplement: Supplementary Figure 3 — Negative control of wholemount human RPE choroid tissue. (A–D) Representative images showing Alexa 546, Alexa 488, merged, and DAPI channels, respectively, in the absence of primary antibodies. For this negative control, no primary antibodies were added. Instead, during the primary antibody incubation step, the tissue was incubated in blocking buffer alone for the same duration as the primary antibody incubation used for the stained tissues. All other staining steps, reagent concentrations, wash conditions, and imaging parameters were identical to those used for the primary stained tissues, as outlined in the Methods. Scale bar: 50 µm. [file Image3.tif]
